# Accelerating Biocatalysis Discovery with Machine Learning: A Paradigm Shift in Enzyme Engineering, Discovery, and Design

**DOI:** 10.1021/acscatal.3c03417

**Published:** 2023-10-26

**Authors:** Braun Markus, Gruber Christian C, Krassnigg Andreas, Kummer Arkadij, Lutz Stefan, Oberdorfer Gustav, Siirola Elina, Snajdrova Radka

**Affiliations:** † Department of Biochemistry, Graz University of Technology, Petersgasse 12/2, 8010 Graz, Austria; ‡ Enzyme and Drug Discovery, Innophore. 1700 Montgomery Street, San Francisco, California 94111, United States; § Moderna, Inc., 200 Technology Square, Cambridge, Massachusetts 02139, United States; ∥ Codexis Inc., 200 Penobscot Drive, Redwood City, California 94063, United States; ⊥ Novartis Institute for Biomedical Research, Global Discovery Chemistry, Basel CH-4108, Switzerland

**Keywords:** biocatalysis, machine learning, enzyme evolution, enzyme optimization, enzyme design, enzyme engineering

## Abstract

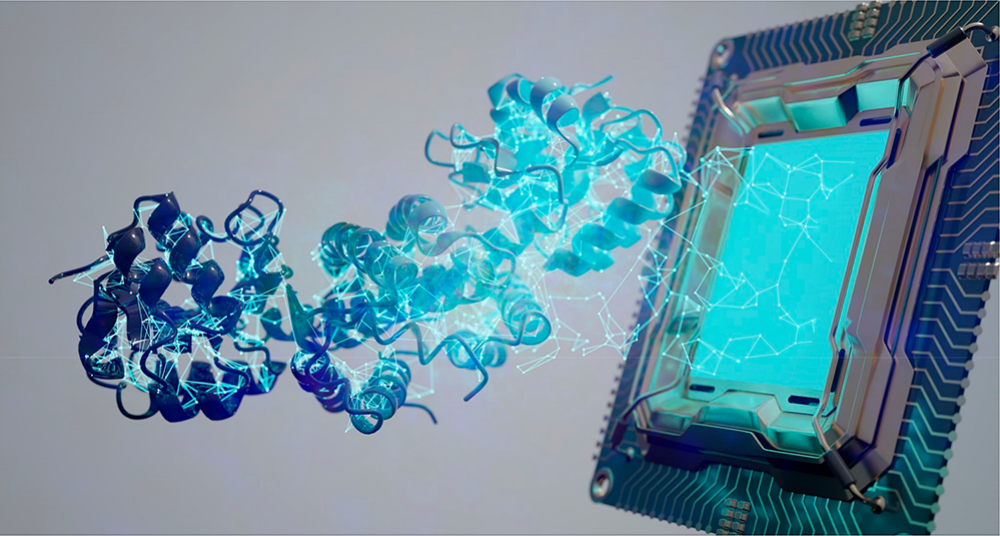

Emerging computational tools promise to revolutionize protein engineering for biocatalytic applications and accelerate the development timelines previously needed to optimize an enzyme to its more efficient variant. For over a decade, the benefits of predictive algorithms have helped scientists and engineers navigate the complexity of functional protein sequence space. More recently, spurred by dramatic advances in underlying computational tools, the promise of faster, cheaper, and more accurate enzyme identification, characterization, and engineering has catapulted terms such as artificial intelligence and machine learning to the must-have vocabulary in the field. This Perspective aims to showcase the current status of applications in pharmaceutical industry and also to discuss and celebrate the innovative approaches in protein science by highlighting their potential in selected recent developments and offering thoughts on future opportunities for biocatalysis. It also critically assesses the technology’s limitations, unanswered questions, and unmet challenges.

## Introduction

**Figure 1:**
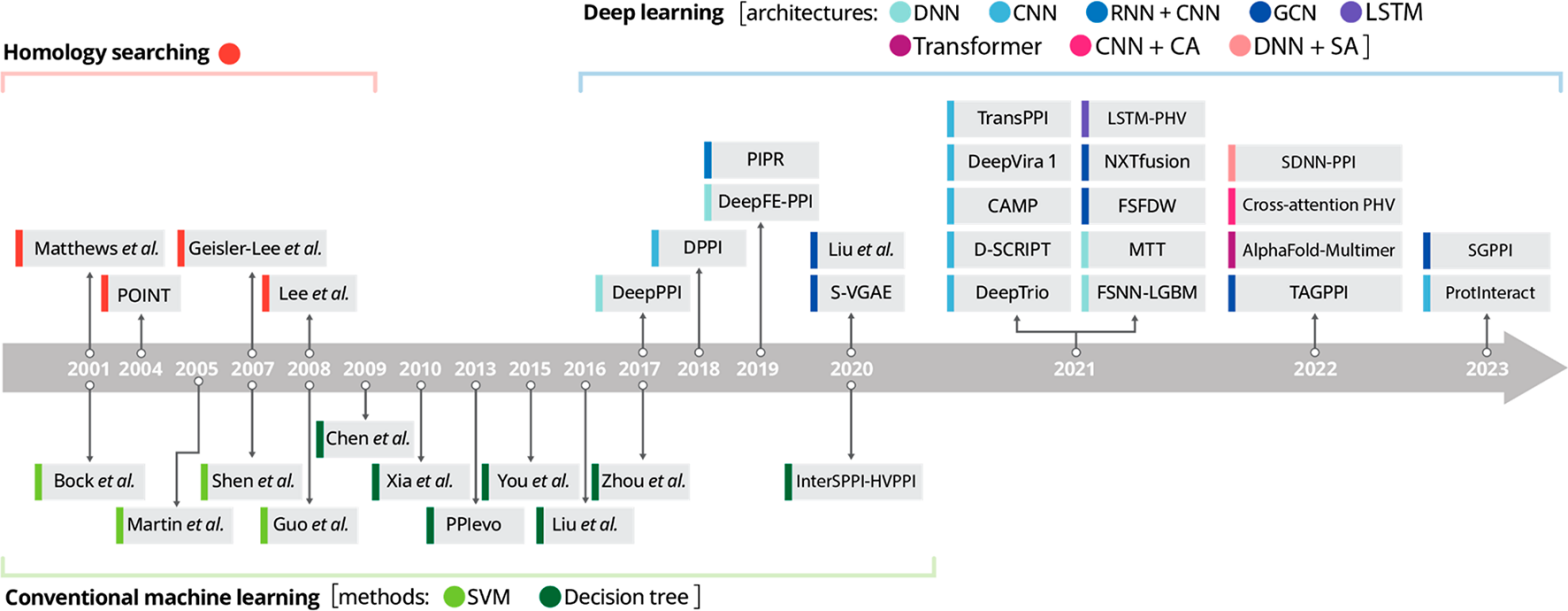
Illustration of the timeline of the typical recent development of ML models visible across various fields but representatively exemplified for the specific domain of studying protein–protein interaction prediction. Figure adapted with permission from ref (7) and extended based on the literature date.^8−13^ Copyright CC BY 4.0 Deed (https://creativecommons.org/licenses/by/4.0/).

Over the last two decades, biocatalysis has secured its position as a standard approach in the chemist’s toolbox when it comes to exquisite specificity and stereoselectivity of chemical transformations, in particular the manufacturing of small molecule active pharmaceutical ingredients (APIs), as well as fine and bulk chemicals.^1^ Such studies, dating back to the early 1900s,^2^ mostly relied on wild-type enzymes, which limited the scope and versatility. With the introduction of directed laboratory evolution came the opportunity to tailor native enzymes to desired process conditions, accessing and improving activity for novel and native substrates, increasing stereoselectivity, and adapting to reaction environments of choice. Compared to attempts of rational enzyme engineering, Darwinian evolution proved more effective in successfully navigating the immense size and complexity of protein sequence space. In parallel, these efforts were greatly aided by advances in DNA sequencing, high-throughput screening, and analytical methods. Nevertheless, the gradual climb up “Mount Improbable” is time- and labor-intensive and comes with the risk of being deceived by false summits. Offsetting some of these risks, computational tools have quickly become invaluable guides in design and analysis of experimental directed evolution workflows (Figure 1, timeline).

Today, *in silico* methods represent an essential component of the design–build–test–learn cycle in enzyme engineering. Promising a faster, cheaper path to high-performance biocatalysts, the combination of bioinformatics and machine learning algorithms offers new tools to potentially locate advanced enzyme engineering starting points and more direct routes to *de novo* and highly engineered high-performance biocatalysts. Multiple excellent reviews and perspectives already summarize applications of this rapidly expanding field.^3−6^

Machine learning comes with the great promise to accelerate enzyme engineering, similarly to the potential that machine learning (ML) and artificial intelligence (AI) have in life sciences.

In everyday conversations and reports, the terms AI and ML are often used interchangeably. These concepts are related, but distinct. AI is the broader concept of machines that are able to perform tasks in ways that we would commonly associate with human-like intelligence. The list of such tasks includes items from categories like problem solving, decision making, and, above all, learning. ML, on the other hand, is a set of computational tools from various paradigms that essentially uses algorithms to describe, analyze, and learn from data. ML algorithms can identify patterns and relationships among enzymes, sequences, active sites, or other aspects of relevant data; make predictions; and help to take actions in order to achieve a specific academic or industrial goal. Noteworthy applications of ML in life sciences are drug discovery, the development of new medical treatments, enzyme design and synthesis, and targeting specific enzymatic properties and abilities.

In terms of concrete machine-learning methods that have been successfully used for the purposes described in this perspective, it is straightforward to note that model uses and architectures have evolved more or less analogously to the typical evolution of the most successful models and architectures in any application of machine learning. Starting from decision trees and support-vector machines around the turn of the century, deep learning took over shortly after 2010, and subsequent developments have followed the typical path from simple deep-net architectures via convolutional neural networks to recurrent neural networks, graph neural networks, and more complicated architectures. Figure 1 has been adapted from ref (7), which details this development for the specific case of studying protein–protein interaction prediction as a representative example for the general evolution of ML models. Since enzyme function is a specific protein property, selecting a protein–protein interaction and the models used there is a good proxy for the field as a whole. A few works using homology searching are included for reference to non-ML methods overlapping with the beginning of the timeline.

In this Perspective, we start with an overview of recent examples that successfully have applied AI and ML for tailoring biocatalysis, focusing specifically on the pharmaceutical industry. Next, we pivot to explaining current and emerging methods in greater depth. Given the still narrow penetration of ML approaches into this field, we explain the fundamentals and showcase the potential of various methods and approaches in enzyme engineering, enzyme discovery, and design. Throughout the manuscript, we discuss a number of relevant subtopics, present instructive examples, and provide our perspective on the implications of the use of ML methods for each problem or application. Finally, we attempt to look ahead to the future of the field and make some recommendations.

## Applications in Biocatalysis and the Pharmaceutical Industry

In enzyme engineering for industrial biocatalytic applications, the desired end points are often slightly different from classical efficiency measures of an enzymatic reaction, often expressed as *k*_cat_/*K*_m_. Because industrial processes aim to operate at high substrate loadings, preferably without product and substrate inhibition, *K*_m_ often becomes irrelevant and more decisive parameters are *k*_cat_ and operational stability. As a rule of thumb, it is been proposed that *k*_cat_ > 1 s^−1^ is desirable for an industrially relevant biocatalyst. Therefore, classical enzyme engineering for use in industrial processes aims to intensify variant-screening conditions each round to bias the evolution toward process relevance. In machine-guided evolution, however, changing reaction conditions at each round need to be taken into account in the modeling and training processes in order to reliably account for any effects in the data that stem from changes in the setup instead of other variables. As a further challenge, enzyme engineering for industrial applications usually seeks to improve multiple properties simultaneously. Typically, high activity and selectivity are performance indicators of choice, often combined with elevated thermostability and tolerance of nonaqueous solvents.

**Figure 2:**
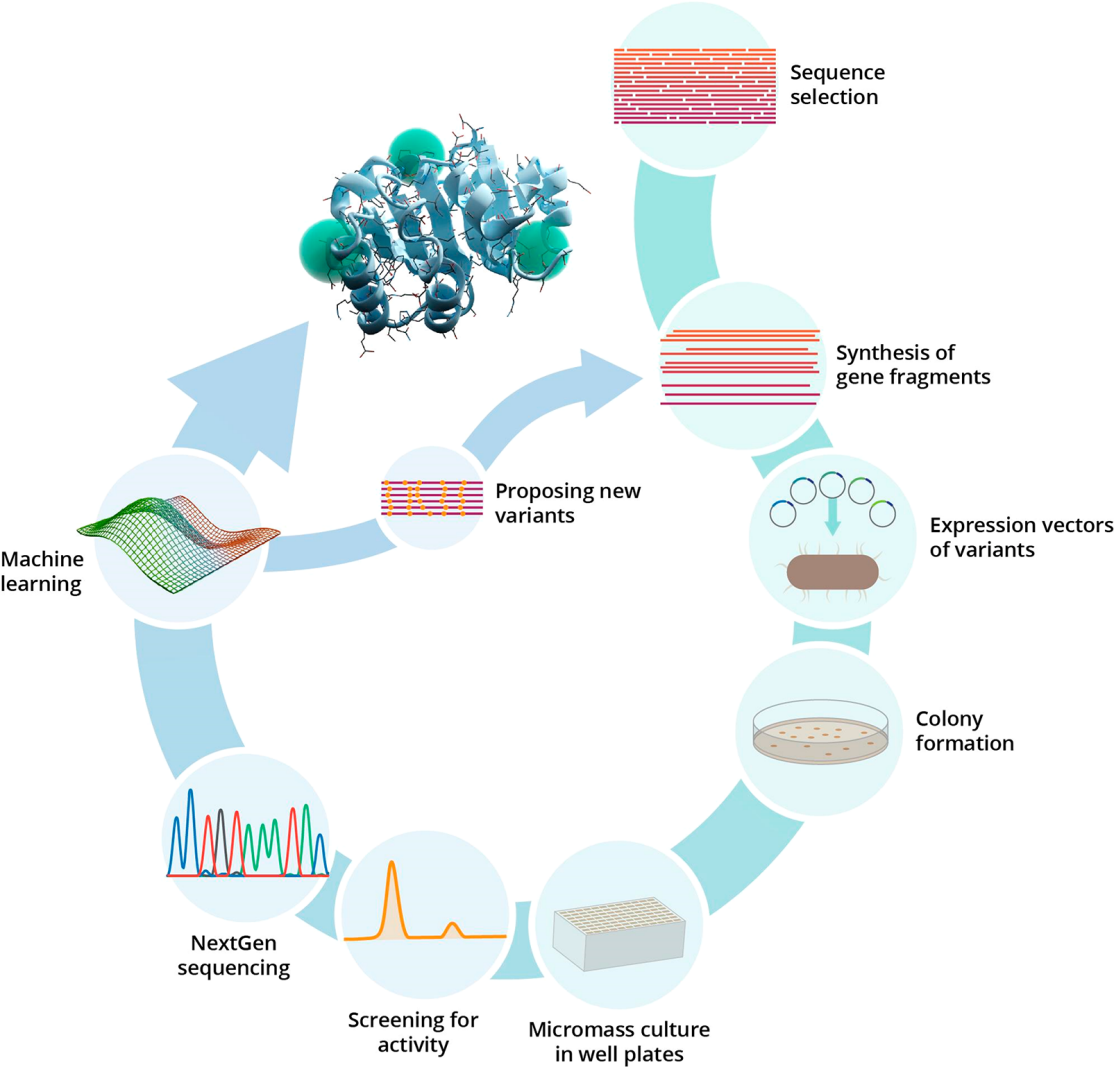
Typical machine learning assisted directed evolution workflow. Reproduced from ref (15). Copyright 2021 American Chemical Society.

**Scheme 1:**
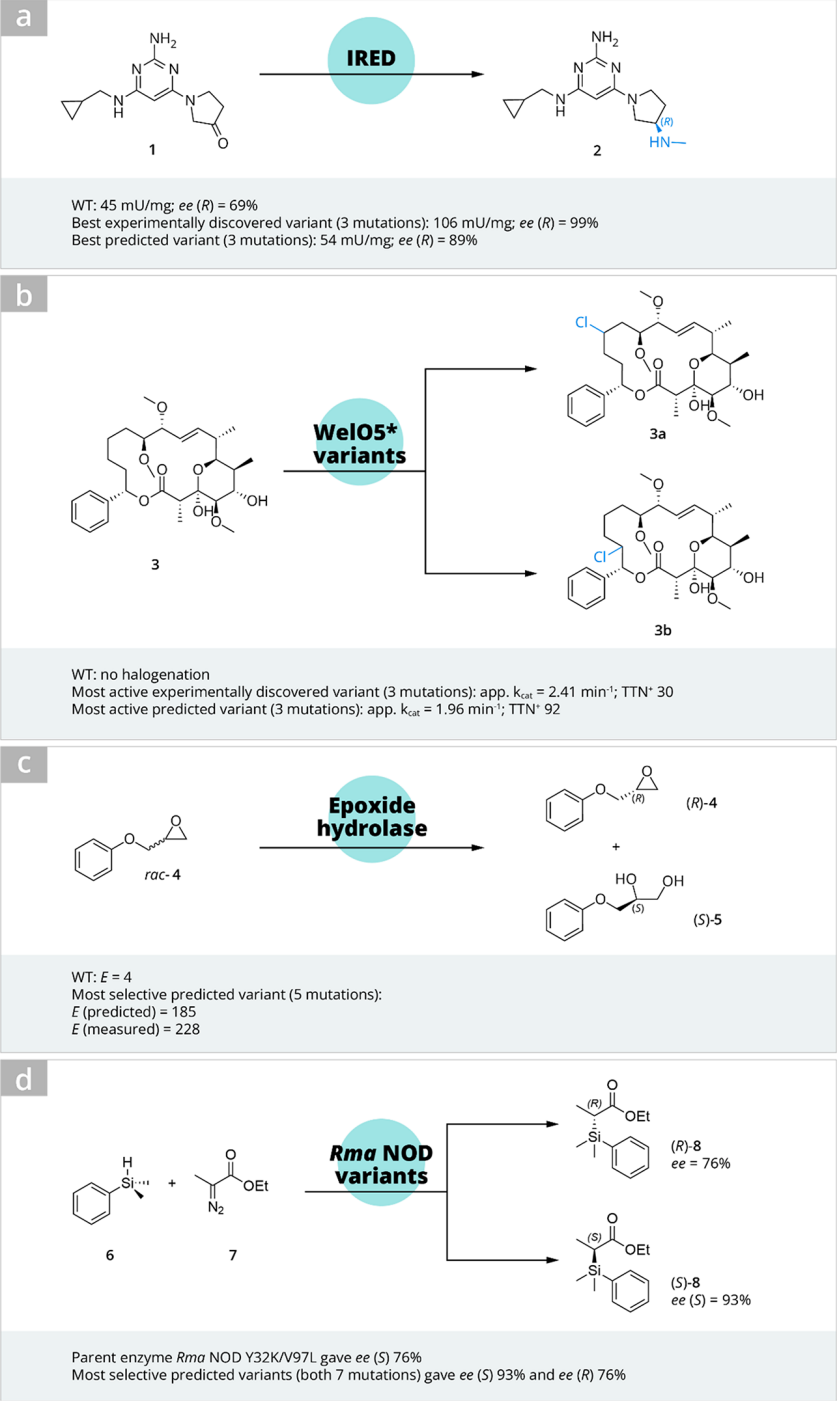
Selected Recent Examples of the Application of AI/ML in Engineering Biocatalysts

At Novartis, scientists embarked on studying machine-directed enzyme evolution to improve the activity and the enantioselectivity of an imine reductase (IRED) catalyzed asymmetric reductive amination of ketone **1** toward an API candidate ZPL389 **2** (Scheme 1).^14^ Their workflow represented a typical machine-directed evolution (Figure 2), starting with selecting a wild-type scaffold and constructing mutant libraries, followed by iterative rounds of screening, analysis, next-generation sequencing, and feeding the ML model for predicting the next round of mutants.

The work aimed to optimize both conversion and enantioselectivity of the reductive amination. Since conversion was determined at a single time point (overnight reaction), it could be argued to reflect both activity and stability components.

The IRED evolution approach relied on a 384-well plate format because the required automation was readily accessible and the team envisioned a relatively large data set and thus high throughput to enable initial (machine) learning. However, homogeneous culturing and enzyme expression from *Escherichia coli* in small volumes inevitably increases the variability of data. To help account for this variability, multiple measurement points for each variant were taken and the true property values were then estimated using Bayesian estimation.^16^ In conclusion, the machine-directed approach yielded variants on par with traditional directed evolution while producing much fewer inactive variants. Reducing the experimental noise in the training data might have further improved the variants generated by the machine-directed approach. For applicability in short project timelines, a “low-N” based methodology^17^ or similar would be highly advantageous and would allow for higher precision of input data. Cases where individual variants could be expressed on a larger scale and even purified for activity and selectivity measurements would significantly increase the quality of data fed into the model. However, the practicality of such an approach strongly depends on the number of variants needed to train a model that is useful for choosing the next round of variants selected for testing.

In the following literature examples, we aim to highlight ML-assisted enzyme engineering work toward biocatalytic applications with relevance to pharmaceuticals. Currently, the studies still focus on methodology, and complete enzyme engineering campaigns toward API manufacturing are yet to emerge.

### Enzyme Activity

As early as 2007, Fox et al.^18^ and Liao et al.^19^ augmented traditional directed laboratory-based evolution with ML strategies. While Fox et al. introduced statistical analysis of protein sequence–activity relationships (ProSAR) to raise the volumetric productivity of a bacterial halohydrin dehalogenase by ∼4000-fold, Liao et al. were able to improve the activity and heat stability of proteinase K toward the hydrolysis of a tetrapeptide by roughly 20-fold using only 95 variants. In both examples, computational analysis of key performance features in initial variants guided the design of subsequent rounds, thereby reducing the number of tested variants while achieving rapid functional gains, highlighting one of the main arguments for leveraging computational tools to increase the efficiency of protein engineering.

Sortase, an enzyme applied in enzymatic bioconjugation (for a recent review, see ref (20)), was a model enzyme in a recent study that compared two sets of training data, one with and one without a known high-activity variant.^15^ Encouragingly, both training sets yielded predictions with comparably improved activities, which were 2.2–2.5× higher than that of the known positive variant.

At Merck & Co, Inc., scientists and engineers were early adopters of biocatalysts and champions of enzyme evolution for API manufacturing. Beyond seminal engineering work with Codexis, including biocatalytic processes in the manufacture of sitagliptin,^21^ islatravir,^22^ and MK-1454,^23^ their quest to accelerate the evolution processes included studies of a broad spectrum of sequence- and structure-based methods (protein GPS, bioluminate and MOE) for mutation predictions in collaboration with ATUM.^24^ The team studied ATA-117, which is a widely applied (*R*)-selective transaminase in the industry. For an initial round of a transaminase evolution, these small (<100 variants) predicted libraries contained 9% and 18% improved variants over the starting point. Combining these mutations resulted in a relatively high number of inactive variants (30% for sequence-based methods and 45% for structure-based methods, respectively). Nevertheless, several variants with 7–9× wild-type activity were also identified.

More recently, Büchler et al. reported an interesting study for engineering a halogenase, which is very relevant to late-stage functionalization in pharmaceutical research.^25^ Wild-type halogenase WelO5* did not catalyze chlorination or hydroxylation of the target substrate soraphen A (**3**, Scheme 1); however several earlier reported variants of WelO5* accepted the substrate **3**. Next, three amino acid positions were chosen for full randomization with theoretical library size of 20^3^ = 8000, out of which 504 unique variants were sequenced and screened. This way, an experimental variant (V81S/A88L/I161P) with a 13-fold increase in total halogenation activity was identified. For the remaining sequence landscape of the library, a Gaussian process model was used to predict higher-activity variants. Four of the seven predicted variants outperformed the first identified variant with up to 16-fold total halogenation activity. Kinetic characterization toward one of the chlorinated soraphen A analogs (**3a**) revealed that although the experimentally discovered variant V81S/A88L/I161P exhibited higher *k*_cat_ (2.41/min) than a predicted variant A88L/I161A (*k*_cat_ 1.96/min), the total turnover number (TTN+) was higher for the latter (92 vs 30), leading to higher product titers with the predicted variant as opposed to the experimental variant. The Gaussian process model prediction clearly reduced the screening effort for the 8000 variant library, which for a 95% coverage with NNK saturation would have required the analysis of ∼100 000 variants.^26^

### (Stereo)selectivity

Engineering the enantioselectivity of enzymes has been a research interest of the Reetz group for a long time. In the earliest adaptation of machine-directed evolution toward improved stereoselectivity of a biocatalyst, an epoxide hydrolase, they applied an adaptive substituent reordering algorithm (ASRA)^27^ and some years later an approach named innov’SAR^28^ to improve the enantioselectivity of the kinetic resolution of *rac*-**4** (Scheme 1). In the latter and newer approach, nine single-point mutants of an epoxide hydrolase, which were originally generated and validated in an experimental enzyme engineering campaign, were used as a training set. The goal was then to predict the enantioselectivity (expressed as *ΔΔG*^‡^ in the study) of all 2^9^ = 512 possible combinations of variants. It was very interesting to see that epistatic and additive effects were predictable using only single-variant training data. The authors observed that Fourier transformation, which innov’SAR applies to generate protein spectra from the numerical encoding of a sequence, improved the model’s prediction performance. After incorporating 28 variants with multiple mutations into training the model, the enantioselectivity (*E*-value) of five arbitrarily selected predicted variants was experimentally measured. Measured values correlated well with the prediction (*R*^2^ value of 0.94). Furthermore, two predicted and measured mutants yielded in much higher *E*-values (>200) toward the resolution of *rac*-**4** than the original best variant from the experimental campaign (*E* = 115).

Machine learning was applied in engineering stereodivergence of a carbene Si–H insertion reaction by the Arnold group (Scheme 1d).^29^ This non-natural reaction was catalyzed (*S*)-selectively (ee (*S*)-**8** = 76%) by the Y32K/V97L variant of a putative nitric oxide dioxygenase from *Rhodothermus marinus* (*Rma* NOD), which was used as a parent enzyme for evolution. During the machine learning-assisted evolution, two sets of amino acid positions were explored. First, four amino acid positions were targeted and combinatorial libraries were built using NDT codons for diverse properties of 12 amino acids at each position. 124 randomly sampled variants from this library were then used as a training set, and subsequently 90 predicted variants for both (*S*)- and (*R*)-selectivity were tested. This first prediction round resulted in 86% ee for (*S*)-**8** (variant VCHV) and 62% ee for (*R*)-**8** (variant GSSG). Those most selective variants were then used as parent sequences for the next round of machine learning-assisted engineering at three new amino acid positions. Variants providing enantiomeric excesses of 93% and 79% of the (*S*)- and (*R*)- enantiomers, respectively, were identified as a result (Scheme 1 d). Importantly, in both of these examples for engineered stereoselectivity, the prediction of epistatic interactions was possible and enabled the discovery of highly enantioselective variants with reduced experimental screening effort.

### Solubility and Stability

Beside activity and enantioselectivity, another important parameter for the optimization of enzymes in industrial settings is their manufacturability, which is the improvement of a protein’s soluble expression and stability under process conditions, such as at elevated temperatures and in the presence of organic solvents. Given the experimental challenges to measure the stability and solubility of large sets of mutants quantitatively,^30^ ML methods offer real advantages to streamline the otherwise expensive and elaborate screening of large mutant libraries. For example, Romero et al. applied a Gaussian process model for T_50_ to maximize the thermostability of cytochrome P450.^31^ The measurement of T_50_ values is a labor-intensive experimental method requiring multiple incubations for each variant and hence is not compatible with high-throughput screening. Modeling enabled the authors to limit their experimental efforts to tens rather than thousands of enzyme variants. Experimental validation of selected candidates confirmed improvements by 5 °C over previously optimized chimeric P450s and 14 °C over the most stable parental enzyme.

Separately, SoluProt is a computational method for the sequence-based prediction of solubility and soluble protein expression in *E. coli*.^32^ Trained using the gradient boosting machine learning technique and TargetTrack database, the authors next benchmarked their algorithm against other solubility prediction methods, showcasing SoluProt’s superior predictive accuracy. The user-friendly and freely available (community-friendly) tool was integrated into the web server EnzymeMiner^33^ for automated mining of novel soluble enzymes from protein databases.

Finally, Repecka et al. trained a generative adversarial network (GAN) model on a wild-type malate dehydrogenase in order to identify functionally improved variants.^34^ While this method is able to “learn” from natural protein-sequence diversity and enables the generation of functional protein sequences, the algorithm also yielded a high proportion of soluble enzymes. Of the experimentally tested sequences, 35% were found to express solubly in *E. coli*, out of which 68% retained measurable malate dehydrogenase activity (24% overall).

In addition to soluble protein expression, enzyme stability during engineering campaigns and under process conditions is a key factor to consider. Multiple studies have shown that engineering enzymes toward higher thermostability also increases their tolerance toward organic solvents; thus, the mechanisms for improving stability somewhat overlap.^35^ For more examples and scientific details, we refer readers to a recent review presenting a perspective on the computational design of stable and soluble biocatalysts.^30^

### Multiparameter Optimization

Simultaneous and efficient optimization of multiple parameters is the ultimate goal toward industrial biocatalysis. Typical features include activity, selectivity, and stability, as well as the alleviation of inhibition. Glucose oxidase was engineered toward higher mediator specificity across broader pH range,^36^ as the optimum pH 5.5 for the wild-type enzyme is not well compatible with applications such as glucose monitoring from physiological samples. For direct biocatalytic applications, it is worth noting that glucose oxidase can be applied as an *in situ* hydrogen peroxide generator when this drives further biocatalytic reactions.

A question encountered in this context of AI-driven enzyme evolution is how well one particular method’s specific applicability will hold up across different enzyme classes. For example, galactose oxidase has at times served as a scaffold to stereoselective alcohol oxidases.^37^ While we can expect a general methodical approach to remain valid for other use cases as well, one may also ask how much of the details in terms of a particular methodology and setup used in the multiparameter optimization of glucose oxidase, for example, would immediately work for galactose oxidases and ultimately for alcohol oxidases.

A step toward generalization of models was recently demonstrated in a transaminase study addressing both activity and stereoselectivity.^38^ Importantly, the generated model could predict variant activity on a different transaminase backbone after retraining on a small additional data set. A methodical approach was taken consisting of (1) the rational design of a mutant library of transaminases (PDB 3FCR) with diverse activities and stereoselectivities; (2) the collection of a standardized, high-quality data set matrixed from 32 variants, the wild-type 3FCR, and 13 pairs of enantiopure substrates; (3) building a predictor for catalytic activity and stereoselectivity; and (4) the prediction and validation of catalytic properties of new variants. After model validation, the authors then looked into extending the prediction to another transaminase, 3HMU, which shares similar active-site architecture to 3FCR. However, updating the predictor with some 3HMU-relevant features was necessary to reach acceptable correlation between predicted and measured values on 3HMU.

### ML for Therapeutic Enzymes

Biocatalysis has made an impact in the synthesis of chemicals and pharmaceuticals over the last years. Beyond catalysis in the world of low-molecular-weight compounds, there is also the opportunity to use enzymes as therapeutics^39^ with various modes of action. Many therapeutic enzymes have been already approved by the FDA and brought to market.^40^ In contrast to small molecules, the use of ML tools for biotherapeutics is still in its early stages. This is probably due to the nature of the enzyme’s application, as additional properties such as safety, formulation and physical and chemical stability need to be addressed in parallel and so far only smaller data sets are available. In this context, ML has a great potential to impact on the optimization of protein properties to accelerate the development timelines and manufacturing cost of biotherapeutics.^41^

## Machine Learning for Enzyme Discovery

### General Considerations

First, we would like to define what we mean by the term *enzyme discovery* in order to distinguish it from the closely related terms enzyme or protein *engineering* as well as *optimization*. For the purpose of this Perspective and the sake of clarity, we would like to define *enzyme discovery* as a process in which either a new protein is discovered that exhibits a new or known enzymatic function or activity or a known protein is shown to possess an enzymatic function or activity that was known from other enzymes, but not for this particular protein. A particular aspect of the latter case is enzyme promiscuity,^42−44^ where the new activity appears in addition to the enzyme’s main function.

In contrast, as soon as any particular protein has been known to be active with regard to a particular enzymatic reaction, further improvement, modification, or mutational analysis of that reaction would thus have to be considered enzyme optimization or engineering instead.

We would like to note here that enzyme discovery is not predictable per se. Despite this marked difference to the processes of engineering and optimization, there are various mechanisms in our arsenal that allow for enzyme discoveries being made while being aimed at something else, be it to some or to a major extent.

### Mechanisms of Enzyme Discovery

One example of such a mechanism is the discovery of new enzymatic functions employing a setup that uses directed evolution in order to optimize a completely different enzymatic function of a set of proteins and their variants.^45,46^ Directed evolution per se is intended to improve a given marker for a protein by mutating and selecting from the “fittest” resulting mutants and is mostly intended for protein engineering. However, enzyme discovery happens during these processes and can be focused on by allowing for new functionality and using further directed evolution to enhance the newfound function.^47^

Another example is the appearance of new enzymatic functionality in the analysis of metagenomic studies.^48^ There, the actual functional analysis may involve challenges, e.g., expression differences in genes found in the overall sequencing effort when they are cloned to other hosts.^49^ On the other hand, recent efforts have led to a growing toolbox, which has ample room for the application of machine learning as well as standard techniques.^50^

A further example is the appearance of a new enzyme function in the study of disease mechanisms,^51^ where the known involvement of a particular protein in causing the disease is suddenly explained by discovering the underlying enzymatic activity. This kind of discovery may appear arbitrary at first, but is made very plausible if not likely in the light of the extensive network of human enzymes and the way their activity is interlinked across multiple different metabolic pathways and diseases.^52^

In summary, enzyme discovery is overshadowed by enzyme engineering in the literature and certainly in practice but must not be neglected due to its potential for exciting new functionality that is waiting to be discovered.

### The Role of Bioinformatics in Enzyme Discovery

In the context of this Perspective, we have to emphasize the various ways in which bioinformatics methods can contribute to enzyme discovery. We already mentioned enzyme discovery as a possible byproduct of a directed evolution effort. While directed evolution is not bioinformatics as such, the computational efforts to aid in this successful method are increasing in amount and sophistication.^53^

That is not all, however. There are several processes that represent very active fields of research in bioinformatics and at the same time could have a drastic impact on enzyme discovery. One such example is protein–protein interaction, where one would like to predict a range of effects that two proteins can have on each other.^54^ For such surface-based interactions, it is natural to also look for smaller molecules that could interact similarly with the protein surface and lay the groundwork for an enzymatic reaction.

An analogous result can emerge from the studies of small molecules (e.g., drug candidates) that bind to the active sites of proteins.^55^ There, one might find a new enzymatic functionality during the experimental validation phase of the corresponding search process. In both these kinds of setups, bioinformatics methods have been very successful in recent years, and machine learning is becoming increasingly important in these computational approaches.^56^

In terms of concrete methods, we would like to stress the importance of machine learning in an open discovery process. In this context, some caveats apply, since it is not straightforward to pinpoint any particular direction for the next scientific discovery. Still, useful strategies follow via an evolutionary picture of phylogenetic and other connections among proteins.^57^ It is the specialization and optimized nature of enzymatic activity as such that makes this kind of relational insight possible.

## Machine-Learning Methods for Protein Engineering

### Protein Representations

A key factor in the success of applying ML to protein engineering is the choice of representation of the protein. Protein representations are essential for ML models to process the complex information encoded in protein sequences and structures. Similar to chemical fingerprints used in cheminformatics, protein fingerprints are employed to represent proteins.

Some common protein representation methods include one-hot encoding, which is a binary representation of protein sequences where each amino acid is represented by a unique binary vector. In other words, the protein sequence is converted into a matrix where the rows represent amino acids and the columns represent the position in the sequence, each column having a 1 at the row representing the amino acid at that position and a 0 otherwise. Property-based representations capture specific physicochemical properties of amino acids, such as hydrophobicity, charge, and size. These representations are mostly residue-based, focusing on individual amino acids and their properties.^58^

Evolutionary-based methods for representing proteins, on the other hand, work on full sequences and incorporate information from homologous sequences.^59^ This approach allows for the consideration of the broader context of the protein sequence, accounting for the relationships between amino acids and their evolutionary history.

**Figure 3:**
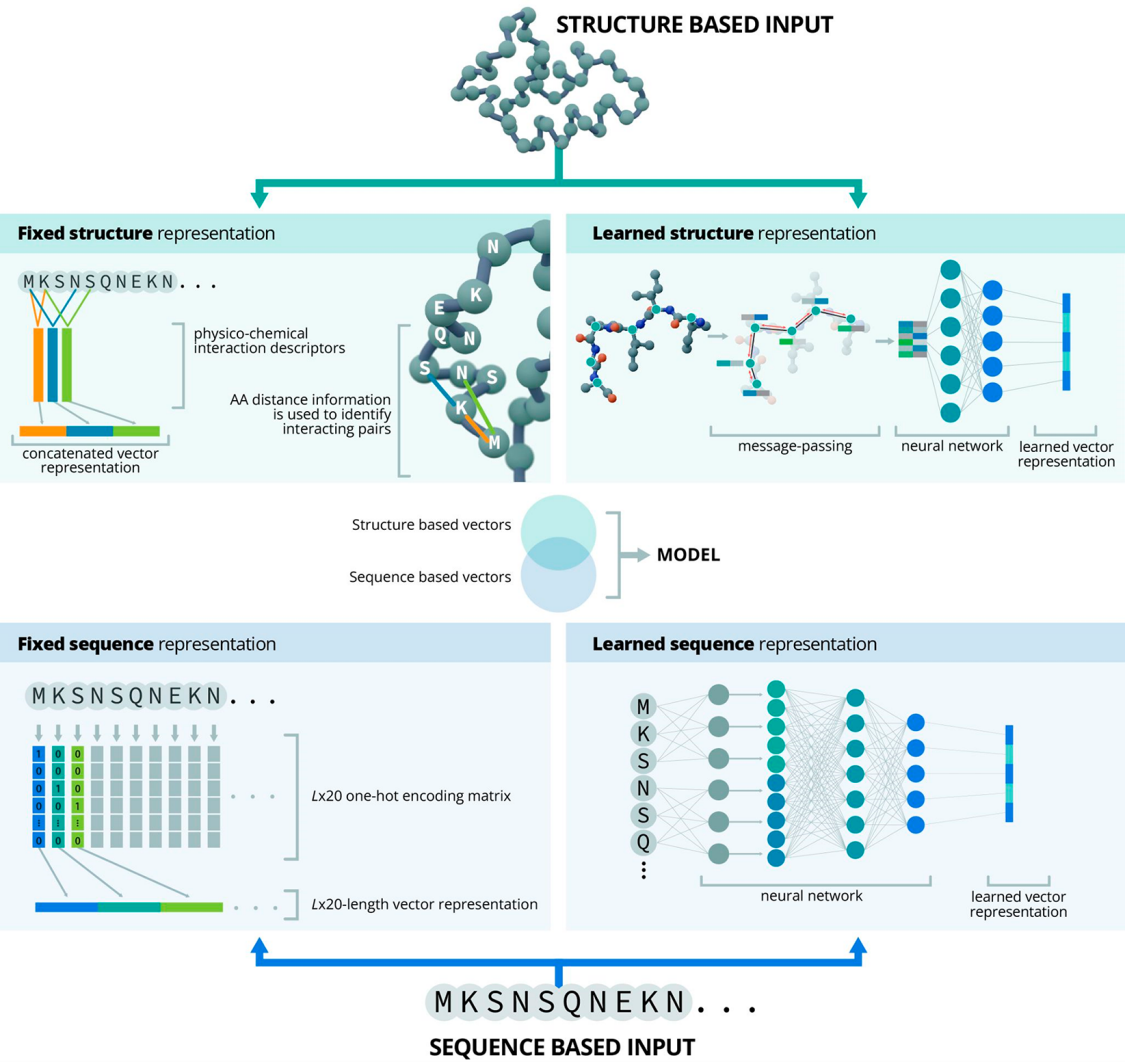
Representation of protein sequences and structures for machine learning. Both sequences and structures can be represented by fixed or learned representations (also known as embeddings). While fixed representations are deterministic and based on inherent sequence or structural features, learned representations, as the name suggests, are often generated by neural networks via unsupervised learning on large unlabeled data sets. Simplified examples of each representation technique are shown. In each case, the final representations are numerical vectors suitable as input for machine learning models. Models can be trained on either just sequence or structural representations, as well as on a combination of both.

In contrast to the so-called fixed representations mentioned above, learned representations, which are generated by advanced methods like large language models (LLMs),^60^ autoencoders,^61^ and other deep-learning models, can learn more abstract and meaningful representations of protein sequences and structures. These models are trained on large amounts of protein sequence and/or structural data to capture complex patterns and relationships within the data, resulting in more information-rich representations. Figure 3 shows examples for the above-mentioned classes of representations.

The main difference between learned representations and fixed representations lies in the fact that learned representations can learn intricate, high-dimensional relationships within protein sequences. Predictive models that are based on such learned representations can potentially extrapolate and make more accurate predictions even in unexplored areas of the sequence space, meaning they can predict the effects of mutations that are not explicitly present in the training data. This feat is hardly achievable using fixed representations. Thus, using learned representations can enhance model performance in various protein engineering tasks, such as protein design, function prediction, and protein–protein interaction prediction.

A common strategy to get sequence-based learned representations is to train LLMs on protein sequence databases to learn the “grammar” of evolutionary and structural information encapsulated in protein sequences.^60^ The resulting protein language models are capable of producing meaningful representations of protein sequences^62^ that could be used for downstream structure prediction or variant-effect-prediction tasks without needing to finetune the models on specific enzyme families or folds.^63^

3D protein structures can similarly be turned into both fixed and learned representations. While fixed structure representations, similar to fixed sequence representations, rely on predefined known properties of the protein structures, such as 3D atomic coordinates, learned structure representations often use deep learning models like graph neural networks (GNNs), which can learn to represent the protein structure in a way that is most useful for the specific task at hand.

The GearNet model published recently by Zhang et al.^64^ uses a relational graph convolutional network to model the interactions between residues in a protein. It also introduces a new concept of edge message passing (as opposed to message passing between nodes of the protein graph) to model different spatial interactions among residues. For more background on message passing neural networks, see Gilmer et al.^65^ In a further step, the group then combined a protein LLM (ESM-1b) with their GearNet model to generate structure-aware protein sequence embeddings.^66^

Detlefsen et al.^67^ investigated how to create meaningful protein representations for various tasks, such as enzyme engineering. They found that the optimal choice of representation depends on the specific task and that different biological aspects of a protein will place different demands on the representations. For instance, some tasks may require a representation that captures the global properties of a protein, while others may need a more detailed view of local properties. This implies that a single protein representation may not be suitable for all tasks and that it is essential to consider the specific requirements of each task when designing a representation.

Interestingly, the authors also discovered that fine-tuning of LLMs can sometimes lead to overfitting and reduced performance. Fine-tuning is often used for models producing learned representations, where additional protein sequences closely related to the engineering target are shown to the model, thereby changing the overall representations produced by the model. This highlights the importance of carefully considering the trade-offs between fine-tuning and using a non-fine-tuned representation when working with protein data.

### Predicting Protein Properties

**Figure 4:**
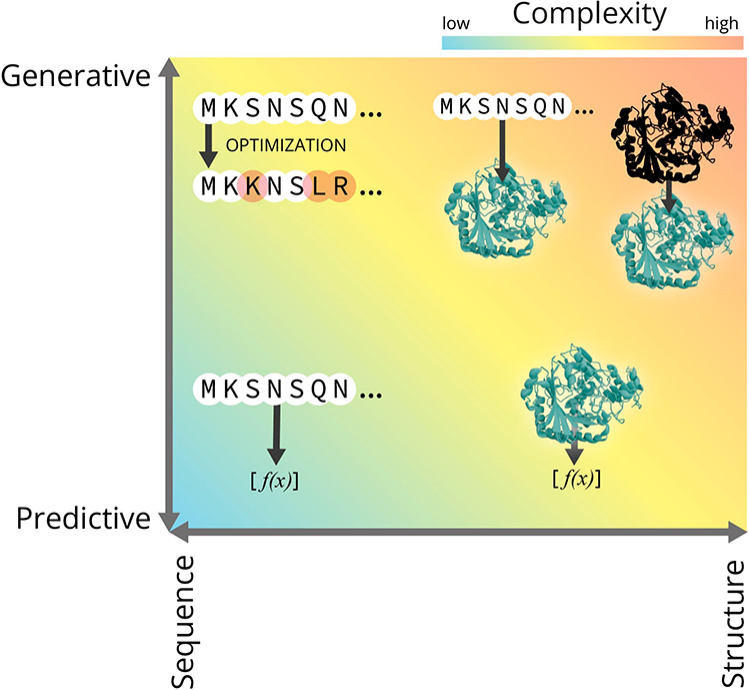
Two main axes of variation for ML models in protein engineering. In any ML-guided protein engineering project, the two basic questions that arise are, first, what are the inputs (protein sequences, structures, or both) and second, what does the model do (predict biophysical properties, generate novel sequences/structures, or both). The more we move toward structure-based modeling as well as generative modeling, the more complex it becomes to both build and operate these models.

Having chosen a suitable protein representation, ML models can be trained to predict protein properties based on the representations, or even generate completely novel protein variants with desired properties (Figure 4). When developing a predictive model, it is generally advisable to start with a simple, non-deep-learning model to ensure there is a signal in the data, *i.e.*, that we are able to predict an experimentally measurable property from either the sequence or the structure of a protein.

Scikit-Learn offers a range of basic ML models that can be used for this purpose, such as random forests, boosting trees, and support vector machines (SVMs).^68^ In Ma et al.,^14^ random forest models were trained to predict the activity and stereoselectivity of variants of an imine reductase. The models were then used to prioritize variants to test experimentally from a large set of novel *in silico* generated variants. The selected variants achieved similar fitness to variants found via classical brute-force-directed evolution. For such straightforward prioritization tasks, non-deep-learning models such as the random forest model can give satisfying results. However, the authors also saw that the model was not able to accurately predict the fitness of out-of-distribution mutants, meaning enzyme variants with combinations of mutations that had not been observed in the training data.

To have better predictive power in such unseen parts of sequence space, or in general to increase model power, deep-learning models can offer a solution. Convolutional neural networks (CNNs),^69^ initially designed for image-prediction tasks, have emerged as a consistently well-performing deep learning tool in protein sequence–function prediction tasks.

Xu et al.^70^ conducted an extensive study on the performance of various ML models as well as the underlying protein representations in accurately predicting protein properties in protein engineering campaigns. After evaluating predictive performance of different deep-learning as well as non-deep-learning models on public and proprietary data sets, they found that CNN models, when trained on amino acid property descriptors, were on average the best-performing models.

Encouraged by these results, Wittmann et al.^71^ added CNNs to their suite of models used in a paper exploring machine learning-guided directed evolution (MLDE). They again evaluated different models and protein representations on their effectiveness in guiding the exploration of the empirically determined fitness landscape of the GB1 protein. Given that sufficient training data points were made available to the models (in this case 384 variants), CNNs consistently ranked among the top-performing models in predicting the fitness of unseen variants.

Another interesting point to mention here is that the authors found the composition of the initial training data to matter a lot in terms of how well the models aided in finding optimal variants. Inclusion of too many unfit variants in the training data hampered model performance. Since in reality most mutational variants of proteins are nonfunctional, and often the fitness landscape of the protein target is not known in advance, the authors propose the so-called zero-shot prediction of the fitness of initial variants. Zero-shot prediction models are capable of predicting protein fitness without any labeled training data specific to the problem. Among the tested methods, EVmutation, mask filling using the evolutionary scale modeling (ESM) protein language model, and triad DDG calculations, were effective in identifying fit GB1 protein variants, with the mask-filling protocol using the ESM model being a notable exception to the general failure of mask filling (which is used to train most current LLM protein models) as a zero-shot predictor.

Gruver et al.^72^ further substantiated the efficacy of CNNs in protein design coupled with Bayesian optimization. They compared three surrogate models (probabilistic predictors of protein function) for protein engineering on synthetic benchmarks and found that CNN ensembles trained directly on primary sequences outperformed Gaussian processes and models built on pretrained learned embeddings. The superior performance of CNNs was attributed to their improved robustness on out-of-distribution data, a critical aspect in protein engineering where the search spaces are often vast and complex.

In conclusion, these studies collectively underscore the potential of CNNs as an effective tool for protein and enzyme engineering.

### Generating New Protein Sequence Variants

For the generation of new variants based on sequence information, variational autoencoders (VAEs),^73^ generative adversarial networks (GANs),^74^ and transformer-based models,^75^ among others, can be used. A prominent example in the chemical space is the use of VAEs to generate new molecules with desired properties.^76^

VAEs have successfully been applied to the protein space as well, creating novel, functional enzymes. Hawkings-Hooker et al.^77^ used a VAE trained on 70 000 luciferase-like oxidoreductases to generate novel functional variants of the luxA bacterial luciferase. While the novel variants showed comparable luminescence to natural variants, they differed in 18–35 mutations to the closest natural protein, showing the capability of VAEs to generate functional variants in unexplored parts of sequence space.

Giessel et al.^78^ used a similar VAE model to generate novel variants of the human ornithine transcarbamylase (hOTC) enzyme. Again, without explicit information about sequence properties, the VAE generated functional variants with on average eight mutations compared to the wild-type hOTC enzyme. The generated variants showed on average improved thermal stability as well as specific activity *in vitro* compared to both the wild-type and a variant based on the consensus sequence from 5000 OTC enzymes. Finally, the authors expressed VAE variants that performed well *in vitro* in HepG2 cells for *in vivo* testing, and while expression levels were *on par* or slightly reduced compared to the hOTC wild-type, specific *in vivo* activity was improved in 7 out of the 12 VAE-generated variants tested.

Having seen that generative models like VAEs are capable of producing functional and even improved novel enzymes simply using sequence-inherent information, the logical next step was to try to bias these models explicitly to generate variants with desired properties. This can be achieved by coupling a model that predicts functional properties of an enzyme to the generative model. The predictive model can guide the generative model toward highly functional regions of (latent) sequence space.

Stanton et al.^79^ combined a denoising autoencoder (a model similar to a VAE) with a Gaussian process regressor that predicts protein properties. Using a multiobjective Bayesian optimization approach, they generated novel red-spectrum fluorescent proteins (RFPs) with improved predicted stability and solvent-accessible surface areas (SASAs). *In vitro* testing of these novel RFP variants showed that the authors’ model produced variants with both higher melting temperatures and brightness compared to any variant in the training data. While this latter application has not yet been validated to result in industrially relevant improvements to enzymes, using a multiobjective optimization approach while factoring in uncertainty in predictions, coupled with a generative model, is in our opinion a highly promising direction to focus on. While classical directed evolution is highly effective at optimizing enzymes for a single property, it is much harder to use it in multiobjective optimization settings.

While generative models have shown that they can indeed come up with functional enzyme variants, it is still hard to predict which of the generated variants will actually fold and function. Paired with the fact that in real-world engineering applications the bottleneck is often the expression and assaying of variants in the lab, metrics to evaluate the quality of *in silico* generated variants before testing them in the lab can be useful to avoid wasting resources on nonfunctional variants. Johnson et al.^80^ evaluated computational metrics to assess the quality of *in silico* generated protein variants.

Two metrics were moderately predictive of enzyme function: ESM-1v likelihood scores and ProteinMPNN scores. On the other hand, neither sequence identity to natural sequences nor AlphaFold2 residue-confidence scores (pLDDT) were found to be predictive of enzyme activity. This indicates that high sequence identity to natural variants or high-quality AlphaFold2 structures do not necessarily guarantee enzyme functionality.

### Bayesian Optimization

As briefly touched on in the examples of Gruver et al.^72^ using ensembles of CNNs and Stanton et al.^79^ using a Gaussian process model, adding an uncertainty estimation to ML models is highly useful to maximize the chances of success in finding high-performing variants. Greenman et al.^81^ did a comprehensive evaluation of various uncertainty quantification (UQ) methods for protein sequence–function prediction.

Having a measure of uncertainty in ML model predictions can help identify regions of the sequence space where the model is underconfident. Following the explore–exploit trade-off, a balance between testing variants with a high certainty of performing well and variants with a high uncertainty of their performance (but potentially in highly desirable regions of the landscape) can be struck. This way, proteins in underexplored regions of the sequence space can be sampled while still continuously testing variants with a high probability of success.

Bayesian optimization is highly effective in striking this balance while being able to also incorporate prior knowledge about the problem, constraints to the search space, and the ability to optimize multiple objectives simultaneously. This has successfully been used in chemical synthesis engineering to reduce the number of experiments required to find the optimal conditions for a reaction^82^ and can similarly be applied to protein engineering.^83,79,84,72,85^

Integrating the aforementioned methods into iterative design-build-test-learn cycles can help in continuously improving protein engineering efforts over rounds of engineering. Using Bayesian Optimization in both candidate selection and experimental design, these cycles enable the systematic exploration of the protein sequence space and facilitate the discovery of novel protein variants with desired properties. By iteratively refining the models based on experimental feedback and adapting the experimental setup to constraints such as batch sizes and screening budgets, the efficiency and effectiveness of the protein engineering process can be enhanced. Greenhalgh et al.^86^ set out to engineer enzymes toward improved activity on acyl-ACP substrates. Using gene shuffling to sample sequence space and Bayesian optimization-guided machine learning to identify optimized sequences, they identified an enzyme displaying twofold higher in vivo fatty alcohol titer than the best natural sequence tested. They performed a total of 10 design–build–test–learn cycles while only testing 10–12 sequences each round. Hu et al.^87^ achieved a 4.8-fold improvement in the selective production of a rhamnolipid congener by optimizing the RhlA enzyme over four design–build–test–learn cycles again leveraging Bayesian optimization for efficient sequence space exploration.

## Machine Learning in *De Novo* Enzyme Design

### Basic Idea Behind *De Novo* Enzyme Design

State of the art machine learning greatly assists the understanding of enzyme structure and function.^88^ The synthesis of highly sought-after products often requires chemical transformations for which no analogs can be found in nature. For such “xenobiotic” chemical transformations, the space of naturally occurring enzymes is inherently a limiting component. The generation of enzymes outside of the evolutionary toolbox requires their design from scratch based on the biophysical principles that guide the desired chemical reaction. This task is called *de novo* enzyme design. Its feasibility was elegantly shown, *e.g.*, by Hilvert et al. in the late 1980s by engineering catalytic antibodies to catalyze a Diels–Alder reaction^89^ or the de Grado and Dutton groups in 1994 with their design of multiheme-containing proteins for redox reactions.^90^ With the development of the physics-based Rosetta software package,^91^ the first novel “xenobiotic” enzymes such as aldolases^92^ or Kemp eliminases^93^ were designed. Yet, even though these enzymes were active, the exhibited activities were below industrially relevant levels and could not be meaningfully improved by computational means alone. Apart from the limited understanding of enzyme biophysics, this failure was in our opinion most likely due to the low accuracy and speed of most methods in computational structural biology and a very limited set of suitable starting structures.

### ML Surrounding *De Novo* Enzyme Design

ML most noticeably impacted computational structural biology in 2021 with the introduction of the highly accurate and fast protein-structure-prediction networks RoseTTAFold^94^ and AlphaFold2,^95^ both of which leverage the power of transformer architectures. Parallel to these advancements, in the earlier years of ML protein design research, generative adversarial networks (GANs) and variational autoencoders (VAEs) were being explored as potential tools for protein design.^67,96^ However, as the field advanced, these methods were superseded by models with more capable architectures, like denoising diffusion probabilistic models (DDPMs). These models, known for their success in text-to-image generation, were able to leverage the “structural awareness” of structure prediction models for highly accurate generative protein-structure modeling.^97−99,99^ Conditioning DDPMs with structural motifs extracted from native enzymes lead to the generation of high-quality protein backbones scaffolding the structural motifs. For the task of designing minimum-energy sequences onto generated protein backbones, models based on geometry aware transformers, such as ESM-IF^100^ and ProteinMPNN,^101^ have greatly outperformed purely physics-based sequence design methods on the metric of native sequence recovery. The geometry-based ProteinMPNN, which is built on a message-passing framework, was further experimentally validated to produce soluble and highly thermostable sequences. For the structure-free generation of protein sequences, deep-learning architectures used in natural language processing (for example GPT) were trained on protein sequence databases to learn the “grammar” of evolutionary and structural information encapsulated in protein sequences,^60^ ESM2.^102^ The resulting protein language models were capable of producing meaningful representations of protein sequences^62^ that could be used for downstream structure prediction or variant-effect-prediction tasks without needing to finetune the models on specific enzyme families or folds.^63^ Generative protein language models like ProtGPT2^103^ and ProGen^104^ were successfully employed to generate novel enzyme variants guided by enzyme-family descriptors. A comprehensive overview of most recent deep-learning based protein design methods can be found at.^105^

### Toward *De Novo* Enzyme Design

Deep-learning methods readily demonstrated their effectiveness for designing protein structure. Whether this translates to an ability to design industrially relevant enzymes *de novo* is not clear yet. The striking improvement that deep learning brought to the speed and accuracy of protein structure modeling is still pending for the task of modeling structure and dynamics of protein ligand complexes. For example, DiffDock, the current state-of-the-art method to predict small-molecule binding, could predict only 38% of protein–ligand complexes in the PDBind database to an RMSD of less than 2 Å.^106^ Yet, fully automated *de novo* design of enzymes will likely depend on deep-learning methods that thoroughly model the behavior of small molecules. We think that the ability of these methods to represent ligands in the context of enzyme active sites at atomic resolution will be essential to account for, *e.g.*, dynamic and electrostatic effects that drive the high catalytic activities found in natural enzymes. Currently, the training of such all-atom models is hampered by a lack of high-quality data sets that contain protein–ligand complexes in conjunction with dynamic and enzymatic information. The training of highly accurate models that can predict small-molecule behavior thus requires meticulous curation of the available experimental data. The MISATO data set by Siebenmorgen et al. is one such example.^107^ The authors refined roughly 20000 protein–ligand complexes from the PDBind data set^108^ using semiempirical quantum mechanics. Next, they computed molecular dynamics trajectories for all complexes, thereby joining high-quality structural information with dynamic descriptors. For the data set SPICE, Eastman et al. computed forces and energies of, in total, 1.1 million conformations for a diverse set of small molecules at the ωB97M-D3(BJ)/def2-TZVPPD level of theory.^109^ We suspect that deep-learning models trained on such high resolution/high level of theory, yet synthetic quality data may represent another route to outperform current physics-based methods in speed and accuracy for the task of modeling the behavior of small molecules in the context of enzyme active sites.

### Promising Ways to Incorporate ML

To date, because of the lack of deep learning methods that accurately depict structure–function relationships for small molecules, *de novo* enzyme design requires combining multiple deep-learning methods with physics-based methods into comprehensive protocols. Yeh et al. recently employed such a combination to design artificial luciferase enzymes.^110^ Using an approach they called “family wide” hallucination, they constructed a diversified library of idealized NTF-2 scaffolds that enclose a binding pocket with shape complementary to their targeted synthetic luciferase substrates. Next, they employed the physics-based RifDock to extract proteins from the scaffold library that could hold the catalytic amino acids in the required catalytic geometry. With their approach, they designed artificial luciferases that, after only one round of site-saturation mutagenesis, exhibited higher selectivity and activity compared to native luciferase enzymes. Albeit successful, this composite approach requires expertise and can be highly error-prone because most deep-learning based sequence- and structure-generation methods were not trained explicitly on enzyme–activity data. An orthogonal yet not “truly” *de novo* approach for designing enzymes was recently described by Lipsh-Sokolik et al.,^111^ in which large fragments of naturally occurring protein structures were successfully recombined to yield highly active xylanases. An in-depth analysis of the thus generated enzymes revealed several metrics that correlated significantly with enzymatic activity. When the authors trained a “simple” ML model to capture the correlated features, they found that it can be used as an activity predictor for their design approaches. Such a strategy would in principle be feasible for any reaction envisioned and thus highly attractive for industrial applications. In our opinion, making *de novo* enzyme design industrially relevant will require rigorous testing of structure–function relationships of enzyme catalysts with iterative design-build-test-learn cycles. Nevertheless, we can imagine *de novo* enzyme design to be a viable addition in the biochemist’s toolbox over the course of this decade.

## Conclusions and Outlook

Biocatalysis and enzyme engineering in particular are multidisciplinary scientific topics involving molecular biologists, biochemists, computer scientists, chemists, and engineers. Communication among technical experts, especially between experimentalists and computational scientists, is of crucial importance to clarify goals and avoid failures in experimental design, data interpretation, and the use of wrong methods. Education on how to effectively communicate across disciplines will play an important role as continuous growth of ML applications in enzyme engineering is to be expected. This aspect goes beyond the technical experts working together on a project, but also reaches out to funding agencies and publishing sphere, where it is difficult to find reviewers with sufficiently broad expertise.

Similarly, the need to not just describe the results but better explain the reasons why a particular method has been chosen and how it compares to other ML methods would be very valuable for the community of readers.

The utilization of more complex computational models for enzyme engineering raises the bar for the level of expertise required to operate these models effectively. Particularly in an industrial setting, the sufficient availability of skilled ML or deep-learning engineers may not always be guaranteed. The application of advanced models necessitates not only a deep understanding of the underlying algorithms but also the ability to fine-tune these models based on the specific task at hand, interpret the results accurately, and troubleshoot any issues that may arise. Furthermore, the development of novel models or techniques requires a high degree of creativity and inventiveness, which is a skill set that may be even rarer. Therefore, while the potential benefits of employing more powerful models are significant, organizations must carefully consider the trade-off. The investment in highly skilled ML engineers to operate and innovate with these models can be substantial, and it is crucial to ensure that the potential gains in predictive power and efficiency outweigh these costs.

In addition, it is critical to have sufficient wet lab capabilities and data infrastructure. As long as fully *de novo* computational design of enzymes is not yet a reality, the generation of high-quality data for training ML models and the experimental validation of newly proposed variants are key components of the protein engineering process. For example, although the biocatalysis community has accumulated a vast amount of enzymatic screening and reaction optimization data over the past decades, it currently is impossible to harness the learnings of all the accumulated knowledge from the current electronic laboratory notebooks, not to mention earlier records. More recently, there are concerted efforts to establish standards in reporting of biocatalytic data. This is critically important because without the ability to generate, validate, and store machine readable data in the lab, the utility of even the most sophisticated computational models becomes severely limited. Therefore, a balanced investment in both computational and experimental resources is essential for the successful future application of ML in enzyme engineering.

Looking at the general landscape of current ML models and the corresponding trends, it is hard to overstate the importance of awareness of the increasing speed of methodological developments in the field. Dominance cycles in the succession of popular models have been shortening, while the potential for general applicability has been increasing. This holy grail of generalizability lies at the heart of ML itself, and is one of the keys to success with these very powerful methods.

It is only fair to expect drastic and even more accelerating developments in the coming years, also for the benefit of enzyme discovery, engineering, and *de novo* design. One distinctive strength of the currently emerging most powerful ML models is the possible integration of many types of different data into extremely large multimodal models^112^ that develop a broader understanding of the underlying mechanisms.

ML has become an integral part of drug discovery, which is constantly expanding with the advent of innovative ML-based discovery tools. In particular, recent advances in generative ML models have facilitated the *in silico* creation^113^ and optimization^114^ of protein binders and small molecules.^106^ On top of modeling binding behavior, predicting biocatalysis also requires a deep understanding of enzymatic mechanisms and corresponding chemical implications, making it “a “tough nut to crack” and one of the most challenging areas for ML.

However, to more consistently apply ML in these fields, several scientific advancements need to occur. There should be stringent requirements for data collection and increased efforts to make data sets, along with corresponding codes, publicly available. Such approaches, such as EnzymeML,^115^ will enhance the reproducibility and reliability of results while also providing a larger, diverse data pool on which ML models can be trained. Additionally, it is crucial for ML models to simultaneously target multiple properties, such as activity, stability, selectivity, and solubility. By considering these parameters concurrently, we can foster a more comprehensive understanding of molecular behavior, enhancing the accuracy of *in silico* designs and predictions. Further incorporating molecular dynamics into ML training can account for the dynamic nature of molecular systems, which is often not captured by static models. This integration could lead to more precise predictive models, bringing us closer to the realistic modeling of molecular behavior.

Despite this complexity, the advancements in data structuring and categorization can pave the way for ML-accelerated biocatalysis. Moreover, to facilitate widespread adoption, ML tools must be made more user-friendly for enzyme engineers, simplifying their utilization in real-world scenarios. In this context, the complexity of enzymology does not pose an insurmountable barrier but rather presents an exciting frontier for future ML advancements. Indeed, by strategically addressing these areas, we can foster a more seamless and effective integration of ML into drug discovery and biocatalysis.
